# Intracardiac thrombus discovered incidentally during respiratory distress in an 18-month-old infant: case report

**DOI:** 10.11604/pamj.2025.50.24.45613

**Published:** 2025-01-13

**Authors:** Youssef Aarjouni, Youssef Absa, Charaf Sayouti, Larbi Dafali, Aziza Bentalha, Salma Echcherif Kettani

**Affiliations:** 1 Department of Anesthesiology and Critical Care, Mohammed V Military Training Hospital, University Mohammed V of Rabat, Rabat, Morocco,; 2 Department of Anesthesiology and Critical Care, The Children’s Hospital of Rabat, University Mohammed V of Rabat, Morocco

**Keywords:** Intracardiac thrombus, pediatric, thrombophilia, case report

## Abstract

Intracardiac thrombi (ICT) are uncommon in the pediatric population and can lead to significant morbidity and mortality. This case highlights a unique instance of a giant ICT in an 18-month-old infant with no notable medical history, contributing to the understanding of hypercoagulable states and their implications in pediatric patients. The patient presented with respiratory distress, characterized by a flu-like syndrome, cyanosis, and hypoxemia. Initial examination revealed lethargy and significant respiratory distress, with imaging showing lobar pneumonia and an echocardiogram revealing a 32 mm x22 mm heterogeneous mass in the left ventricle. Further investigations confirmed the mass was a thrombus, attributed to deficiencies in proteins C and S. The patient was treated with dobutamine to enhance contractility, furosemide for diuresis, and heparin for anticoagulation. There was a favorable progression, with gradual weaning from oxygen and complete resolution of the thrombus by day 15. This case underscores the importance of recognizing hypercoagulable states in pediatric patients with ICT. It suggests that medical management can be an effective alternative to surgical interventions. Moreover, it emphasizes the need for further research to establish optimal treatment protocols for pediatric ICT, particularly regarding the risks associated with thrombus size and ventricular function.

## Introduction

Intracardiac thrombi (ICT) are rare in children, but when they do occur, they can result in significant morbidity and mortality. Over the past few decades, reports of ICT incidence have increased, particularly in a hypercoagulable state [1]. In the pediatric population, the majority of ICT occurs in the context of a provoking risk factor, such as low cardiac output syndrome, arrhythmias, and/or hypercoagulable states. We report the case of a patient admitted to the intensive care unit for respiratory distress, in whom trans thoracic ultrasound revealed a giant intracardiac thrombus. Through this case study, we delve into the main etiologies of ICT in the pediatric population and the therapeutic options available to optimize patient outcomes.

## Patient and observation

**Patient information:** this is an 18-month-old male infant (GT.) with no significant medical history, who was admitted to the emergency department due to respiratory distress that had been worsening for one week before admission.

**Clinical findings:** the clinical examination revealed a lethargic and hypotonic infant, exhibiting tachypnea at 60 breaths per minute and an oxygen saturation of 89% on room air. The infant showed signs of marked respiratory distress, characterized by suprasternal and intercostal retractions. Hemodynamically, the infant was tachycardic for their age, with a heart rate of 175 beats per minute, a blood pressure of 105/65 mmHg, and a capillary refill time of less than 3 seconds. Pulmonary auscultation noted a decrease in breath sounds, particularly in the right apical region, along with bilateral crackles in the mid-lung fields. Cardiac auscultation did not reveal any additional heart sounds.

**Timeline of current episode:** on 23/04/2024: chest X-ray was performed, 24/04/2024: a transthoracic echocardiogram performed on the patient's bed, 25/04/2024: thoracic angioscanner, 26/04/2024: thrombophilia analysis.

**Figure 1:**
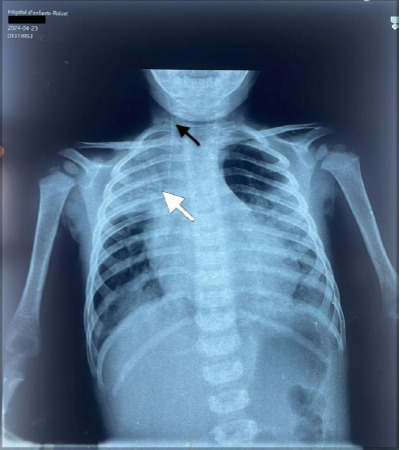
front chest X-ray showing: lobar pneumonia (black arrow); right internal jugular central line catheter (white arrow)

**Figure 2:**
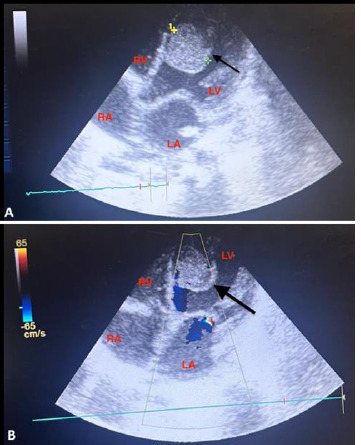
A, B) apical section of cardiac ultrasound showing a giant mass in the left ventricle

**Figure 3:**
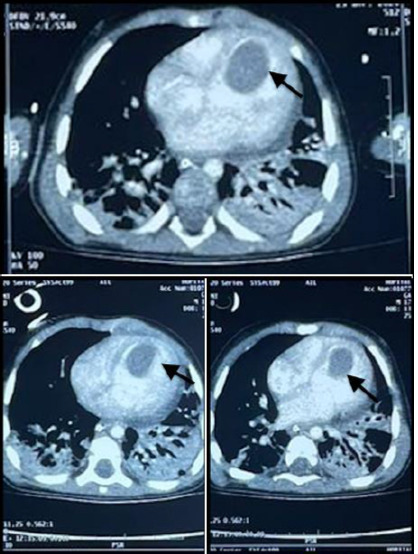
axial section of thoracic angioscan showing a giant thrombus in the left ventricle

**Diagnostic and assessment:** in light of the acute respiratory distress, an etiological investigation was initiated in the emergency department, where a chest X-ray revealed right lobar pneumonia (Figure 1). After transferring the patient to the pediatric intensive care unit and stabilizing their condition a transthoracic echocardiogram performed at the bedside revealed a heterogeneously echogenic mass measuring 32 mm by 22 mm in the left ventricle, attached to the latero-apical wall, with associated hypokinesis but without ventricular dilatation or congenital heart defects (Figure 2). Faced with this intra-ventricular mass, two possible diagnoses were considered: a tissue mass or a thrombus. Further investigations included a thoracic angioscanner and a thrombophilia analysis. The angioscan confirmed the thrombotic nature of the mass (Figure 3). The thrombophilia panel indicated deficiencies in protein C and protein S, with protein C activity at 20% (normal range: 40-92%) and protein S activity at 40% (normal range: 65-130%). Other coagulation factors were within normal limits.

**Diagnosis:** the primary diagnosis was hypercoagulability due to deficiencies in protein C and protein S, leading to a thrombus in the left ventricle.

**Therapeutic interventions:** initially, the treatment involved preparatory measures, including establishing a 22G peripheral intravenous line, positioning the patient in a semi-upright position, administering high-concentration oxygen therapy via a mask at a flow rate of 15 L/min, and placing a bladder catheter to monitor urine output. Symptomatic treatment for acute heart failure was initiated with dobutamine at a dose of 10 µg/kg/min to enhance contractility, along with furosemide at a dose of 1 to 2 mg/kg/day to reduce congestion. For the etiological treatment, broad-spectrum antibiotics were started upon admission, consisting of ceftriaxone at a dose of 50 mg/kg/day in combination with azithromycin at 10 mg/kg/day for pneumonia. Regarding the thrombus, a medical management approach was chosen, implementing an unfractionated heparin protocol that included a loading dose of 75 units/kg administered intravenously as a bolus, followed by a maintenance dose of 25 units/kg/hour via continuous intravenous infusion. This was adjusted based on coagulation tests, specifically targeting an activated partial thromboplastin time (aPTT) of 1.5 to 2.5 times the normal range, with anticoagulation therapy planned for an initial duration of 10 days.

**Follow-up and outcome of interventions:** the patient´s condition worsened on day -2 with respiratory distress, necessitating orotracheal intubation and mechanical ventilation. There was a gradual improvement, evidenced by an enhanced PaO_2_/FiO_2_ ratio, reduced oxygen requirements, and decreased basilar crackles, along with effective diuresis of 1-2 cc/kg/h. On day -7, a follow-up echocardiogram revealed a thrombus that was resolving, with a size reduction. After a successful weaning trial, the patient was extubated and placed on low-flow oxygen via nasal cannula. By day -10, the heparin therapy was discontinued, and oral anticoagulation with apixaban 2.5 mg twice daily was initiated for a duration of 3 to 6 months.

**Informed consent:** the parents gave their approval for the publication.

## Discussion

Intracardiac thrombi can lead to significant morbidity and mortality in any patient group. While management strategies are well-established for adults, there is a lack of comprehensive information regarding their management in the pediatric population [1]. In adults, the most common cause of ICT is dilated cardiomyopathy followed by myocardial infarction, with an incidence of 45% and 61.9% respectively [2]. In pediatric patients, the main cause has not yet been clearly identified. Most studies have concentrated on underlying hematologic abnormalities, such as deficiencies in protein C, protein s and/or mutations of Factor V Leiden, which may make children more susceptible to ICT [3]. Furthermore, both pediatric and adult patients who have undergone the Fontan procedure appear to have an elevated risk. The Fontan physiology, along with its detrimental effects on the liver, seems to predispose these patients to a hypercoagulable state, which may include deficiencies in protein C, protein S, and elevated Factor VII [4]. In our case, the etiological investigation was in favor of a coagulation disorder, i.e, a deficiency of proteins C and S, which were below normal values. Decreased concentrations of protein C are associated with an increased risk of venous and, occasionally, arterial thromboembolism [5]. Protein C deficiency can be either congenital or acquired. Temporary acquired protein C deficiency may occur due to conditions such as disseminated intravascular coagulation, liver dysfunction, heart failure, respiratory distress syndrome, sepsis, and malignancy. In the case we presented, we examined the predisposing factors for cardiac thrombus and identified acquired protein C and S deficiency due to sepsis, which was associated with lobar pneumonia. Clinical and laboratory findings supported the diagnosis of sepsis. A meta-analysis involving 119 adults found that treatment with heparin was linked to more favorable survival odds compared to surgical therapy or thrombolysis [6]. The optimal treatment for pediatric patients with ICT remains undefined. Various treatment options have been reported, including observation, systemic anticoagulation, thrombolysis, and thrombectomy. Case reports indicate that some premature infants with ICT have been treated with urokinase [7], tissue plasminogen activator (tPA), recombinant tissue plasminogen activator [8], and thrombectomy [9].

Overall, the management approach is often tailored to the individual patient's condition and circumstances. In our case, our patient received medical therapy by heparin infusion. In a retrospective review conducted in 2007 involving 31 pediatric patients with ICT [1], medical therapy resolved the condition in 19 out of 30 patients (63%). Only one patient had surgery for a thrombectomy. Embolization occurred in 4 patients (13%), with one death due to a cardiac thrombus. Mortality was higher in patients with dilated cardiomyopathy, linked to the underlying condition rather than ICT itself. Smaller ICTs tended to resolve with medical treatment, while larger ones were more likely to cause embolization. Intracardiac thrombi morphology did not affect embolization risk, and having multiple ICTs was not associated with embolization. All patients who experienced embolization had impaired ventricular systolic function, while none with normal function did [1].

## Conclusion

Pediatric intracardiac thrombi (ICT) are most commonly diagnosed in patients with hypercoagulable states, dilated cardiomyopathy, and those who have undergone Fontan operations, with a notable male predominance. The optimal treatment for these patients is not yet well-defined; however, medical management is an effective alternative to surgical thrombectomy. Smaller ICTs tend to resolve with medical therapy, while larger thrombi carry a greater risk of embolization. There is a significant mortality rate associated with pediatric ICT, particularly for those with left ventricular thrombi or concurrent ventricular systolic dysfunction. To better understand treatment options, prospective multicenter trials are essential for evaluating the efficacy of specific anticoagulant and thrombolytic agents in this patient population.
